# Artificial selection on reproductive timing in hatchery salmon drives a phenological shift and potential maladaptation to climate change

**DOI:** 10.1111/eva.12730

**Published:** 2018-12-03

**Authors:** Michael D. Tillotson, Heidy K. Barnett, Mary Bhuthimethee, Michele E. Koehler, Thomas P. Quinn

**Affiliations:** ^1^ School of Aquatic and Fishery Sciences University of Washington Seattle Washington; ^2^ West Fork Environmental Tumwater Washington; ^3^ Seattle Public Utilities Seattle Washington

**Keywords:** artificial selection, fisheries management, life history evolution, Pacific salmon, phenology

## Abstract

The timing of breeding migration and reproduction links generations and substantially influences individual fitness. In salmonid fishes, such phenological events (seasonal return to freshwater and spawning) vary among populations but are consistent among years, indicating local adaptation in these traits to prevailing environmental conditions. Changing reproductive phenology has been observed in many populations of Atlantic and Pacific salmon and is sometimes attributed to adaptive responses to climate change. The sockeye salmon spawning in the Cedar River near Seattle, Washington, USA, have displayed dramatic changes in spawning timing over the past 50 years, trending later through the early 1990s, and becoming earlier since then. We explored the patterns and drivers of these changes using generalized linear models and mathematical simulations to identify possible environmental correlates of the changes, and test the alternative hypothesis that hatchery propagation caused inadvertent selection on timing. The trend toward later spawning prior to 1993 was partially explained by environmental changes, but the rapid advance in spawning since was not. Instead, since its initiation in 1991, the hatchery has, on average, selected for earlier spawning, and, depending on trait heritability, could have advanced spawning by 1–3 weeks over this period. We estimated heritability of spawning date to be high (*h* ^2^ *~*0.8; 95% CI: 0.5–1.1), so the upper end of this range is not improbable, though at lower heritabilities a smaller effect would be expected. The lower reproductive success of early spawners and relatively low survival of early emerging juveniles observed in recent years suggest that artificial and natural selection are acting in opposite directions. The fitness costs of early spawning may be exacerbated by future warming; thus, the artificially advanced phenology could reduce the population’s productivity. Such artificial selection is known in many salmon hatcheries, so there are broad consequences for the productivity of wild populations comingled with hatchery‐produced fish.

## 1 INTRODUCTION

The ability of animal populations to respond and persist in the face of myriad human‐induced environmental changes is a key concern for scientists, natural resource managers, and those who rely on fish and wildlife for their livelihoods (Badjeck, Allison, Halls, & Dulvy, 2010; Dolan & Walker, 2006). In the face of rapid and often unpredictable environmental change, maintaining or increasing resilience—the capacity of individuals, populations, and ecosystems to adapt and persist—is a common goal of resource management and conservation (Allen, Cumming, Garmestani, Taylor, & Walker, 2011; Gunderson, 1999; Quinlan, Berbés‐Blázquez, Haider, & Peterson, 2016; Walker, Holling, Carpenter, & Kinzig, 2004). Pathways for adaptation include phenotypic plasticity (i.e., behavioral, morphological, and physiological changes at the individual level), range shifts, and evolution (Bernhardt & Leslie, 2013; Parmesan & Yohe, 2003). However, because environmental changes and resulting biological responses are uncertain, the specific traits, populations, or species most likely to persist under future conditions are seldom known (Webster et al., 2017). Consequently, management strategies to maximize resilience often focus on genetic, phenotypic, and species diversity, and the connectivity of populations and habitats. Indeed, benefits of maintaining diversity at multiple scales are widely recognized (Schindler, Armstrong, & Reed, 2015) and include increased abundance and reduced year‐to‐year variability in productivity (Hilborn, Quinn, Schindler, & Rogers, 2003; Schindler et al., 2010). On the other hand, human activities including some management and conservation strategies may unintentionally erode the diversity on which resilience relies (Carlson & Satterthwaite, 2011; Webster et al., 2017).

Human‐induced evolution can affect the diversity and resilience of populations and has been observed in many taxa (e.g., fishes (Heino, Díaz Pauli, & Dieckmann, 2015), mammals (Douhard, Festa‐Bianchet, Pelletier, Gaillard, & Bonenfant, 2016)), in response to such influences as harvest (Allendorf & Hard, 2009), climate change (Bradshaw & Holzapfel, 2006), and pollution (Medina, Correa, & Barata, 2007). Many traits can be affected including growth rate (Conover & Munch, 2002), age at maturity (Olsen et al., 2004), aggression (Sutter et al., 2012), and phenology (Quinn, Hodgson, Flynn, Hilborn, & Rogers, 2007). Human activities—harvesting (e.g., fishing and hunting) in particular (Hendry, Farrugia, & Kinnison, 2008)— can impose strong directional selection resulting in dramatic changes in phenotypic traits with consequences for population dynamics and ecological interactions (Darimont et al., 2009; Palkovacs, Kinnison, Correa, Dalton, & Hendry, 2012).

In addition to harvest, artificial propagation of species for subsequent release to join wild populations can also exert selection and spread maladaptive traits (Derry, 2019; Frankham, 2008). If propagated individuals are later integrated with wild breeders, these trait changes may spread in the broader population, and if the trait changes are maladaptive in the wild, then conservation efforts may undermine the population's productivity or persistence (Baskett & Waples, 2013; Gering, 2019). The approach is particularly common in anadromous fishes where propagation methods are well established, and freshwater habitat loss or degradation often limits populations (Lorenzen, 2005). Indeed, billions of juvenile Pacific salmon (*Oncorhynchus *spp.) are released from fish hatcheries around the Pacific Rim each year (Ruggerone, Peterman, Dorner, & Myers, 2010), to increase harvest opportunities, supplement depleted wild populations, or prevent extinction (Naish et al., 2007). These fish experience different regimes of selection on a number of traits, including reproductive timing, a highly heritable trait in salmonids (McLean, Bentzen, & Quinn, 2005; Quinn, Peterson, Gallucci, Hershberger, & Brannon, 2002; Tipping & Busack, 2004). In some hatchery salmon populations, reproductive timing has been intentionally altered through selection to temporally separate hatchery from wild runs or to enhance the efficiency of the hatchery operations (Crawford, 1979). In other cases, hatchery managers tend to spawn early arriving fish because they are uncertain how many fish will eventually return (McLean et al., 2005), or there are practical challenges to capturing fish for breeding later in the season (e.g., high stream flows). Either or both processes can cause unintended advances in spawning timing (e.g., Flagg, Waknitz, Maynard, Milner, & Mahnken, 1995; Quinn et al., 2002). Early return may reduce fitness by exposing adults to higher water temperatures (Quinn & Adams, 1996) that can result in prespawning mortality (Bowerman, Roumasset, Keefer, Sharpe, & Caudill, 2018). Earlier spawning also advances hatching and emergence of juveniles, potentially leading to a mismatch with environmental conditions or prey resources (Quinn, 2018). Moreover, the salmon that return to spawn over the course of the season commonly differ in body size, in‐stream life span, and reproductive allocation (Doctor & Quinn, 2009; Hendry, Berg, & Quinn, 1999); thus, timing is a very important trait with direct and indirect connections to many aspects of salmon ecology and life history.

**Figure 1:**
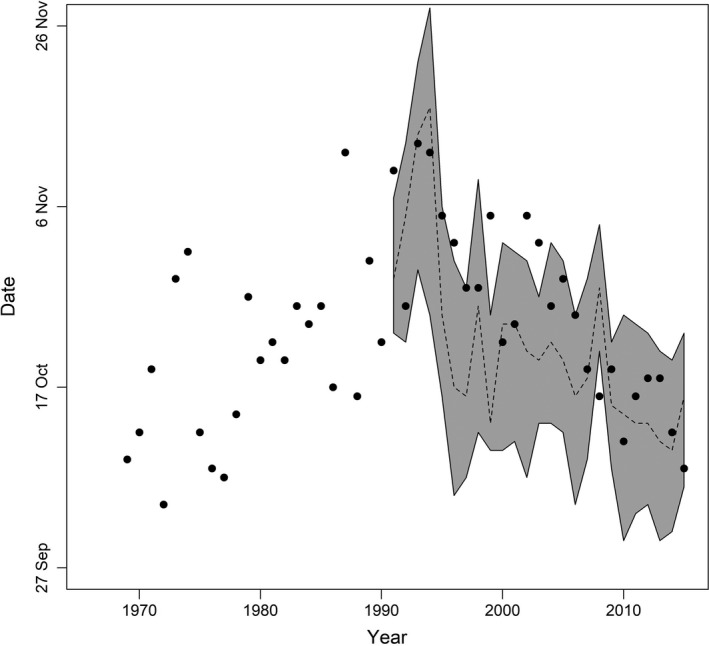
Dates of natural and hatchery Cedar River sockeye salmon spawning, 1969–2015. Points show median date of natural spawning in the Cedar River based on multiple float surveys each year; dashed line shows median date of hatchery spawning; the shaded area spans 25%–75% completion dates of hatchery spawning (see *Salmon Timing Data* in the Methods section for details)

**Figure 2:**
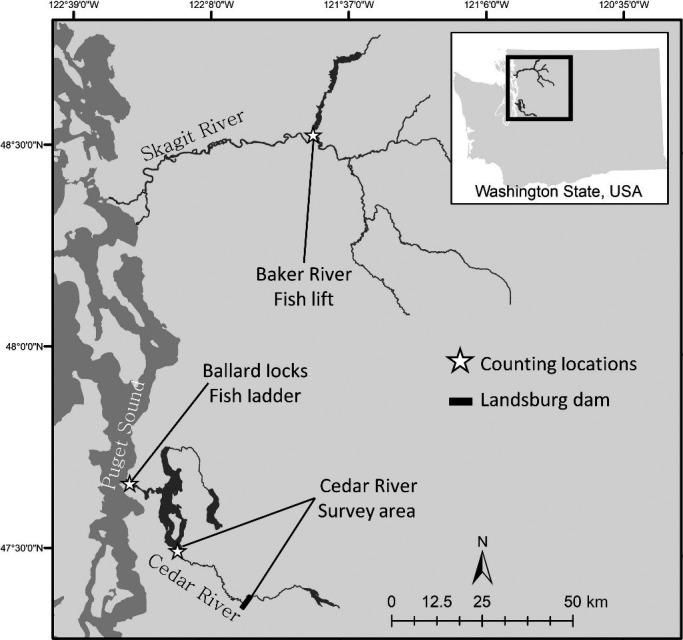
Study region showing the Skagit River and Lake Washington watersheds with sockeye salmon enumeration locations identified

Given the prevalence of salmon hatcheries, their potential influence on migratory and reproductive timing, and the importance of phenology in adaptation to climate change, an interaction between these two selective forces seems probable. In this study, we examine a potential case of such counteracting selection in a population of sockeye salmon (*O. nerka*) that has undergone marked changes in reproductive timing over the past four decades and has been supplemented with hatchery propagated fish as an increasing proportion of the total run since the early 1990s (Figure 1). Prior to 1991, the average escapement to the Cedar River, Washington, USA, (Figure 2) was ~213,000 with no hatchery contribution. In the decades since, the average run size has declined while an increasing proportion of returning adults have been captured for spawning in the hatchery (1990s: 87,000 escapement, 8% spawned in hatchery; 2000s: 77,000 escapement, 11% spawned in hatchery; since 2010:45,000 escapement, 28% spawned in hatchery). Meanwhile, between the early 1970s and early 1990s, natural reproduction by sockeye salmon in the Cedar River appeared to be shifting later in the year, consistent with an effect of local environmental change (e.g., Warren, Robinson, Josephson, Sheldon, & Kraft, 2012) during this period (i.e., increasing water temperatures and decreasing late‐summer flow). In the mid‐1990s—approximately coincident with the initiation of a hatchery supplementation program—this pattern apparently reversed and spawning became progressively earlier in the year. Despite annual hatchery supplementation and an absence of targeted fishing since 2006, the population has declined in abundance in recent years. Because naturally spawned and hatchery‐produced fish interbreed in both the hatchery and the river, there is concern that unintended selection in the hatchery may have contributed to the recent trends in reproductive timing and abundance in the integrated population.

Our overall goal was to describe and explain the patterns of migration and reproductive timing in Cedar River sockeye salmon, testing two hypotheses that might explain them. First, we considered that changes might be phenotypic responses to environmental variation in marine or freshwater habitats (e.g., water temperature and river discharge: Hodgson, Quinn, Hilborn, Francis, & Rogers, 2006). To do so, we compared the patterns of salmon migration and spawning timing to a variety of data sets on conditions along the migratory corridor from the coastal ocean to the spawning stream, including a comparison to the patterns in another Puget Sound sockeye salmon population, Baker Lake. Second, we assessed the potential role of directional selection from hatchery operations. To do so, we estimated the heritability of spawning timing based on data from this system and from estimates of other salmonid species. We then determined whether hatchery operations have been selective, relative to spawning in the river. Finally, we modeled the extent to which the observed changes in timing could be explained by the strength of selection and heritability of timing, and conclude by discussing the potential fitness consequences of artificially altered reproductive phenology for this population, given the regional environmental conditions.

## 2 METHODS

### 2.1 Study system

Lake Washington, WA, USA, is a large, natural lake near Seattle, WA, that has been substantially altered over the past century by human development. Today, the primary input is the Cedar River, which enters from the south and contributes over 50% of the annual inflow (Arhonditsis, Brett, Degasperi, & Schindler, 2004); the lake drains to the marine waters of Puget Sound through the Lake Washington Ship Canal (Figure 2). Prior to the construction of the Ship Canal and the Hiram M. Chittenden Locks (commonly, and hereafter, called the Ballard Locks), and diversion of the lower river reaches in the early 20th century, the Cedar River did not flow into Lake Washington; rather, the lake drained through the now dry Black River (Edmondson, 1991). Because sockeye salmon typically require lakes for juvenile rearing, it is generally thought that the Cedar River had few if any sockeye salmon prior to hydrological modifications of the Lake Washington system (Darwin, 1917). Lake Washington sockeye salmon are thought to be primarily descendants of fish from Baker Lake, Washington, a tributary to the Skagit River located ~120 km north of the Cedar River that were stocked in the Cedar River and other tributaries between 1934 and 1944 (Figure 2; Ames, 2006; Spies, Anderson, Naish, & Bentzen, 2007). Consequently, we also obtained and analyzed data from the Baker Lake system for comparison with the marine entry timing of the Lake Washington fish, as they might be affected by common oceanic factors. Although sockeye salmon spawning also occurs on Lake Washington beaches and in other tributaries, the Cedar River has produced on average more than 85% of all returns since adult counting efforts began in the 1960s. Since 1991, a portion (2%–61%; mean 14%) of returning Cedar River sockeye have been collected using a weir in the river for spawning in a hatchery and their offspring released back to the river as fry to complete the rest of their lives naturally. Hatchery fish are not externally marked, and no effort is made to separate hatchery and natural origin fish. Both hatchery and naturally spawned fish typically spend one year rearing in the lake and two or (less often) three years at sea, and thus achieve a total age of four or five years before returning to complete their life cycle.

### 2.2 Environmental data

Cedar River flow data were obtained from USGS gaging stations located at river kilometer (rkm) 2.6 near Renton, and rkm 32.8 below the Landsburg diversion dam; daily average discharge was available for all study years (1969–2015). Water temperature data along the migratory route (Lake Washington, Lake Union (a small lake between Lake Washington and Puget Sound), and the Ship Canal) were obtained from King County (https://green2.kingcounty.gov/lake-buoy/) and the University of Washington (Edmondson, 1994). Temperatures were typically recorded monthly or biweekly, in which case observations were averaged by month. Monthly temperature data were available for the Ship Canal nearly continuously since 1975 and range from the surface to 5 m depth, while in Lake Washington observations began in 1965 and range from the surface to 60 m. Daily surface water temperatures recorded at a marine site along the migration route into Puget Sound (Race Rocks, British Columbia) were obtained from racerocks.com and are available for all study years.

We summarized flow and temperature data into potential covariates of timing based on their general influence on sockeye salmon timing (Hodgson & Quinn, 2002) or the Cedar River population in particular (Ames, 2006). For Lake Washington water temperatures, we calculated seasonal means (spring: March– May, summer: June–August, autumn: September–November) and annual maxima for each site and depth. For marine water temperatures at Race Rocks, we calculated monthly mean temperatures during the typical period of sockeye salmon migration, May – July. For Cedar River flows, we calculated (a) the mean discharge in the months when most spawning occurs (September through November), (b) the extent of early spawning season high flow events (defined as the number of days between 1 August and 30 September when discharge exceeded the long‐term 75th percentile), and (c) cumulative discharge increase during September (sum of all positive day‐to‐day differences in flow).

### 2.3 Salmon timing data

Sockeye salmon returning to Lake Washington enter freshwater at the Ballard Locks which separates the Lake Washington Ship Canal from the marine waters of Puget Sound. Since 1972, state and tribal fisheries personnel have conducted daily counts of sockeye salmon passing through the locks and/or the fish ladder during an index (12 June–31 July) period that captures on average >95% of the run (i.e., salmon returning to the basin as a whole), with the great majority destined for the Cedar River. Expansion factors are used to account for proportions of the run that occur at night or during uncounted locking cycles (Ames, 2006). Although imperfect, these daily visual counts were very similar to estimates from hydroacoustic methods (Thorne, 1979) and are considered unbiased with regard to the hypotheses addressed here. Counts have ranged from 22,159 to 530,063 per year and have been consistently low since 2007 (Pre‐2007 mean: 261,440; 2007–2015 mean: 75,397). This stage, when the salmon transition from marine to freshwater environments, we hereafter refer to as arrival which is separated in space and time from entry onto the spawning grounds and breeding.

In addition to the timing of arrival in freshwater, the other life history event of primary interest was the timing of spawning in the Cedar River. This could not be directly observed at the population level and so was estimated from a two‐step process, detailed below. Briefly, surveys of live salmon in the river yielded an annual estimate of occupancy, which was the basis of an estimate of when those salmon entered the Cedar River, and from that we estimated the timing of spawning. Surveys of live adult salmon in the Cedar River have been conducted by Washington Department of Fish and Wildlife (WDFW) or other agencies using standardized methods since 1969 (Ames, 2006). An index reach extending from rkm 6.8 upstream to the Landsburg Diversion Dam (rkm 35.1), the upper limit of sockeye spawning, has been surveyed since 1969. Occasional surveys of the lower section of the river (below rkm 6.8) documented few fish and little spawning activity prior to 1987, but the use of this reach by sockeye salmon increased dramatically in recent decades (Timm & Wissmar, 2014). Thus, since 1987, survey counts have been reported for all available habitat (rkm. 0.0–35.1) in addition to the traditional index reach. The inclusion of the lower reaches in more recent years was not expected to bias our analysis because few fish were present in this reach prior to the late 1980s, and because fish spawning in the lower river tend to enter later, which should lead to a delay in observed timing—the opposite of the recent trend. To summarize timing of entry into the Cedar River, survey counts were converted to estimated daily counts through linear interpolation (Barnett, Simmons, & Peterson, 2013). Interpolated daily counts of sockeye salmon for 1969–2012 were obtained from prior reports on counts of live Cedar River sockeye salmon (Cascade Environmental Services Inc. 1995, Barnett et al., 2013) and updated through 2015 with field data from WDFW. As in previous reports, five years (1970, 1973, 1974, 1975, and 1990) were excluded from analysis because too few surveys were conducted to be reliable (Barnett et al., 2013).

Although survey counts of live salmon allow for interannual comparison of timing of occupancy, they do not allow for evaluation of selection on spawning timing within a year, because individual fish may be observed multiple times throughout the spawning season. We therefore used a simple accounting model based on interpolated daily in‐river abundance and estimated in‐stream life span to calculate the number of fish dying each day. Then, the estimated number of deaths was added to the change in live fish between days to yield the number of new fish entering the river. Mathematically,

$${\text{deaths}}_{t}={\text{entering}}_{t-\text{stream}\text{.life}}$$

and

$${\text{entering}}_{t}{\;\text{= live}}_{t}{\;\text{- live}}_{t\;\text{- 1}}{\;\text{+ dead}}_{t}$$

Because of observation error (i.e., error estimating live fish from stream surveys) and process error (i.e., variability in true stream‐life), the model can produce negative estimates of entering salmon which result in oscillations as the estimates are used in the calculation of future deaths. To accurately calculate descriptive statistics for the distribution of entries, we stabilized such oscillations by fitting a cumulative normal distribution to the cumulative distribution of modeled entries using ordinary least squares; these fitted distributions were used for further analysis of entry timing. The temporal distribution of spawning in the river was then estimated by offsetting entry timing by the average number of days that fish are in the river before completing spawning. Cedar River sockeye mature while holding in Lake Washington for several months prior to river entry (Newell & Quinn, 2005; Newell, Fresh, & Quinn, 2007) and then typically initiate spawning relatively soon after entering the Cedar River. Tagging studies in both the river and broodstock held at the hatchery suggest this delay averages slightly more than one week (Ames, 2006; WDFW, unpublished data).

The arrival timing of Baker Lake sockeye salmon, the ancestral population of the Lake Washington run, was used for comparison with the Ballard Locks data on arrival of Cedar River salmon to indicate possible shared marine influences on timing. The Baker Lake fish are captured in a trap approximately 88 river km upstream from marine waters and are counted and transported daily above two hydroelectric dams. Similar to the Lake Washington populations, Baker Lake sockeye arrive primarily during June and July, but do not spawn until the fall. Daily counts of transported sockeye were obtained from the WDFW and the Skagit River System Cooperative for the years 1965–2016. Prior to 1992, Baker Lake sockeye salmon were severely depleted, directed fishing was negligible, and so the counts at the collection facility represented essentially the entire run. The population expanded in recent years and was exposed to some fishing, so from 1992 to 2016, daily counts were corrected for commercial and sport catches in terminal areas (i.e., Skagit Bay and the Skagit River). For this period, daily catches were assigned trap arrival dates by combining the fishing location with a estimate of travel time to the collection site based on tagging studies, are assumed to remain constant between years (Personal communication, Peter Kairas, Skagit River System Cooperative, September 29, 2016). No attempt was made to correct for any harvest of Lake Washington or Baker Lake sockeye salmon in distant fisheries; interception by coastal fisheries is minimal because the Puget Sound runs are earlier than the much more abundant runs to the Fraser River and not sufficiently numerous to merit dedicated fisheries (Starr & Hilborn, 1988). Data were not collected on the spawning timing of this population so they were only used for comparison of arrival timing.

### 2.4 Hatchery operations data

Comparisons between reproductive timing of hatchery‐spawned and naturally spawning fish were used to assess artificial selection on timing by hatchery practices. In the hatchery, all females were checked every few days and those with eggs free from the connective tissue were euthanized and the eggs removed. At this stage, they would be expected to spawn had they been in the river, and so the dates of hatchery spawning were compared to the estimated dates of spawning in the river without any adjustment. The average time from capture to spawning in the hatchery is around eight days; given the similar water temperatures experienced by hatchery and naturally spwaning fish during this period, we believe it unlikely that the delay between river entry and spawning varies substantially based on location (i.e., hatchery vs. in‐river). Daily counts of eggs taken were obtained from WDFW for all years of hatchery operation (1991–2015). All hatchery origin fish embryos were exposed to controlled thermal shifts during embryonic development that induced a permanent set of marks on their otoliths, unique to each group, that can be examined in adult salmon after death (Volk, Schroder, & Grimm, 1994). These marks not only indicated that the fish was produced in the hatchery, but in many years specific marks were also applied based on the timing when their parents were spawned (early, middle, or late in each season). For fish returning between 2005 and 2012, a sample of otoliths was collected during each hatchery spawning event (*N* = 9,571 over all years), and for these years, we compared the timing of spawning in the parental and offspring generations to estimate the genetic control over timing. It should be noted that salmon spawning takes place more or less throughout the season, and thus, the emergence of fry is similarly broad. It is impractical to separately mark the embryos from each day of spawning and then keep separately the associated lots of emerging fry. So, the embryos of fish spawned in the early, middle, and late parts of the run were combined and given the same mark. Even within these groups, they did not all emerge on the same day and so were held briefly until the rest of that group emerged and could be transported to the river for release. Thus, the returning adults were assigned a specific date of spawning but it could only be related to their parents’ timing group (early, middle, or late).

### 2.5 Analysis of long‐term trends in phenology

We calculated median arrival timing from daily counts at the Baker Lake outlet trap, and at the Ballard Locks, river entry timing from interpolated daily counts of sockeye salmon in the Cedar River (because counts were not made daily), and egg take dates (i.e., spawning of females) in the Cedar River hatchery by calculating daily cumulative counts, dividing these by the annual total at each site, and taking the first date that exceeded 50% of the total as the median. We then employed an information theoretic model selection approach to evaluate the shape, magnitude, and influence of environmental covariates on trends in median timing for Lake Washington entry (i.e., passage of the Ballard Locks), entry into the Cedar River, and arrival at the Baker River trap.

Because migration and reproductive timing in salmonids have ostensibly evolved to maximize fitness given prevailing environmental conditions, long‐term change in such conditions may also explain contemporary evolution or plastic changes in these traits (Crozier, Scheuerell, & Zabel, 2011; Quinn & Adams, 1996; Warren et al., 2012). Water temperature and stream flow influence reproductive phenology in many salmonid species, including sockeye salmon throughout their geographic range (Hodgson et al., 2006). Changes in temperature and flow regimes have also been implicated in altered migration timing of other sockeye salmon populations (Crozier et al., 2011; Major & Mighell, 1967). Trends in timing that are not explained by environmental change may indicate that some other process is responsible. To partition these sources of variability in phenology, sixteen candidate models (Table 1) were fit to time series of median timing of Cedar River entry Baker River and Ballard Locks arrival using the glm(general linear model) function in the “stats” package in R.

**Table 1:** Candidate models for evaluating trends in median arrival of sockeye salmon to the Baker Lake system, reflecting entry into freshwater there; the Ballard Locks, reflecting entry into the Lake Washington system; and the Cedar River, where most Lake Washington salmon are produced

ID#	Break point?	Environ. covariate	Formula
1a	No	N/A	~*α*
1b	No	1	~*α* + *β* *E*_1_
1c	No	2	~*α* + *β* *E*_2_
1d	No	Both	~*α* + *β*_1_E_1_ + *β*_2_ *E*_2_
2a	Yes	N/A	~*α* + *β* *R*
2b	Yes	1	~*α* + *β*_1_ *E*_1_ + *β*_2_ *R* + *β*_3_ *E*_1_ * *R*
2c	Yes	2	~*α* + *β*_1_ *E*_2_ + *β*_2_ *R* + *β*_3_ *E*_2_ * *R*
2d	Yes	Both	~*α* + *β*_1_ *E*_1_ + *β*_2_ *E*_2_ + *β*_3_ *R* + *β*_4_ *E* *_1_* * *R* + *β*_4_ *E*_2_ * *R*
3a	No	N/A	~*α* + *β* *Y*
3b	No	1	~*α* + *β*_1_ *Y* + *β*_2_ *E*_1_
3c	No	2	~*α* + *β*_1_ *Y* + *β*_2_ *E*_2_
3d	No	Both	~*α* + *β*_1_ *Y* + *β*_2_ *E*_1_ + *β*_2_ *E*_2_
4a	Yes	N/A	~*α* + *β*_1_ *R* + *β*_2_ *Y* + *β* *_3_* *R* * *Y*
4b	Yes	1	~*α* + *β*_1_ *R* + *β*_2_ *Y* + *β*_3_ *E* *_1_* + *β* *_4_* *R* * *Y* + *β*_5_ *R* * *E*_1_
4c	Yes	2	~*α* + *β*_1_ *R* + *β*_2_ *Y* + *β*_3_ *E* *_2_* + *β* *_4_* *R* * *Y* + *β*_5_ *R* * *E*_2_
4d	Yes	Both	~*α* + *β*_1_ *R* + *β*_2_ *Y* + *β*_3_ *E* *_1_* + *β*_4_ *E*_2_ + *β* *_5_* *R* * *Y* + *β*_6_ *R* * *E*_1_ + *β*_7_ *R* * *E*_2_

Note

Α: intercept; *β*_x_: regression coefficients; *E*_1_: best environmental predictor of timing identified using OLS regression (for Cedar River, natural logarithm of cumulative September flow increase. For Baker River and Ballard locks, mean May temperature at Race Rocks, BC); *E*_2_: second best environmental predictor of timing (for Cedar River, mean autumn surface temperature in Lake Washington. For Baker River and Ballard locks, mean July temperature at Race Rocks, BC); *R*: a categorical regime variable distinguishing between periods before and after an estimated break point; *Y*: year, capturing residual temporal trends after accounting for environmental change

All models evaluated included combinations of stationary or linearly changing median migration dates with one or two potential environmental covariates. For each site, the two most highly correlated environmental covariates with timing were included. Each model was also tested for a break point; optimal breaks were identified by sequentially fitting the models using all possible years. The break point returning the lowest AIC (Akaike's information criterion) was then used for model comparison. For each site, model performance was compared using AIC which balances model fit (likelihood) with model complexity (number of parameters). The model with the lowest AIC was considered the most parsimonious, the relative support for candidate models was evaluated by comparing AIC between models, and the relative likelihood of each model was compared by calculating AIC weights (i.e., exp(−0.5 (AIC_best _‐ AIC_i_)).

In addition to this examination of trends in median dates, we also calculated the 10%, 25%, 75%, and 90% completion dates to qualitatively explore changes in run duration and to make comparisons between sites. Correlations between timing reference points at different locations or different populations may indicate a shared underlying driver of timing (e.g., marine conditions for arrival timing of Baker River and Lake Washington populations). Conversely, a lack of a relationship may indicate that timing is more strongly influenced by population‐specific factors or local environmental signals. Pearson correlation coefficients were calculated between pairs of timing reference points in order to identify the strength and direction of any relationships.

### 2.6 Heritability of spawning timing in cedar river sockeye

All the progeny of adult sockeye salmon spawned in the hatchery from 2004 through 2011 had their otoliths permanently marked by exposure of the embryos to a series of thermal shifts during development. These marks were specific to the periods in the fall when the parents had been spawned (early, middle, and late). These juveniles entered Lake Washington in the years 2005 through 2012, and the great majority returned three years later (i.e., 2008 through 2015). When these returning adults were spawned at the hatchery, otoliths were collected after the fish were killed for spawning from a consistent subsample, revealing whether the fish's parents had been spawned early, middle, or late season four years earlier, as well as its age. These data allowed us to calculate, for each timing group, in each year, the average spawning date for the parental and offspring generations.

Because younger Pacific salmon often spawn later in the season than do older salmon (Quinn, 2018), we analyzed these ages separately. We standardized both generations to zero mean and unit variance, and performed a linear regression of offspring mean timing against parental timing for each age at return. The slope coefficients of these regressions can be interpreted as the realized heritability in spawning timing (Hard, Bradshaw, & Holzapfel, 1993).

### 2.7 Artificial selection on spawning timing

To estimate the direction and strength of selection on spawning timing imposed by hatchery operations, we developed a simple evolutionary model based on the breeder's equation, which has been previously utilized in studies of changing salmon spawning timing (Abadía‐Cardoso, Anderson, Pearse, & Garza, 2013; Crozier et al., 2011; Quinn, Unwin, & Kinnison, 2000). The equation is commonly written as:

$$\Delta Z=h^{2}S$$

Where *Z* is the population mean for a trait of interest and the change in *Z *over one generation equals the product of the heritability of the trait, *h* ^2^, and a selection differential on that trait, *S*. Linear selection differentials measure the difference in trait means between the original population and the selected population, typically the portion of the population surviving to reproduce. In the case of hatchery selection in an integrated population, this raw selection differential overstates the strength of selection because fish that are not spawned in the hatchery can nevertheless produce offspring. Egg‐to‐fry survival is substantially higher in the hatchery setting than in the wild, and *S* can therefore be weighted by this fitness advantage to estimate the effective strength of any selection imposed by the hatchery. For each year of hatchery operation, a raw linear selection differential was calculated as the difference in mean spawning date between fish spawned in the hatchery and the total population (calculated as the mean of hatchery timing and natural timing weighted by the proportion of the return spawned in each setting). These values were then penalized by the proportional egg‐to‐fry survival advantage of being spawned in the hatchery instead of spawning naturally ((hatchery survival – natural survival)/hatchery survival). Survival in the hatchery was estimated from the total number of eggs spawned (mean fecundity, determined empirically for this population, times number of females spawned) minus the number of dead embryos removed during standard culling procedures. The survival of embryos in the river was estimated from the total number of eggs potentially spawned (mean fecundity and number of females), and the number of fry migrating from the river into Lake Washington estimated in annual operations based on regular trapping throughout the spawning season (Kiyohara, 2017). These estimates were not available annually, and so an average value was calculated based on 10 years with available data (0.83). Final selection differentials were then the product of the raw differentials and the egg‐to‐fry survival advantage. Expected change in spawning timing (∆*Z*) in the next generation was determined by the breeder's equation. Uncertainty exists in model parameters including stream‐life, the time between river entry and spawning, the hatchery survival advantage, and the heritability of timing traits. A Monte Carlo sensitivity analysis was therefore conducted to explore the impact of parameter uncertainty on the direction and strength of trait change resulting from hatchery selection.

For all parameters, the Monte Carlo procedure took 10,000 random draws from a broad uniform distribution intended to encompass all plausible values. While area under the curve (AUC) escapement estimation for the Cedar River uses a fixed 15‐day stream‐life estimate based on tagging (Ames, 2006), studies from many other salmon populations (Perrin & Irvine, 1990) and anecdotal observations in the Cedar River (Ames, 2006) suggest that early arriving salmon live longer than those arriving later. There is also some evidence that average Cedar River stream‐life may be declining in response to spatial shifts in spawning activity or increased rates of prespawning mortality (Ames, 2006). The sensitivity analysis, therefore, explored the effect of average stream‐life as well as the influence of declining stream‐life within years (i.e., early arrivals live longer than late arrivals) and between years (i.e., stream‐life is declining over time). The Monte Carlo procedure was conducted three times, with high, moderate, and low levels of spawning timing heritability (*h* ^2^). Table 2 shows the range of values used in the sensitivity analysis, and the point estimates considered to be best estimates for each model parameter.

**Table 2:** Summary of Cedar River sockeye salmon arrival model parameters

Parameter	Monte Carlo Range	Point estimate
Initial stream‐life	14–22 days	18 days
Final stream‐life	50%–100% Initial	11 days
Mean stream‐life	Calculated	14.5 days
Stream‐life trend	−4 to 0 days	−2 days
Spawning delay	5–11 days	8 days
Hatchery egg‐to‐fry %	Calculated	93%
Natural egg‐to‐fry %	Calculated	17%
Hatchery advantage	0.6–0.95	0.83
Heritability	Fixed	0.3, 0.5, 0.83

## 3 RESULTS

### 3.1 Analysis of long‐term trends in phenology

**Figure 3:**
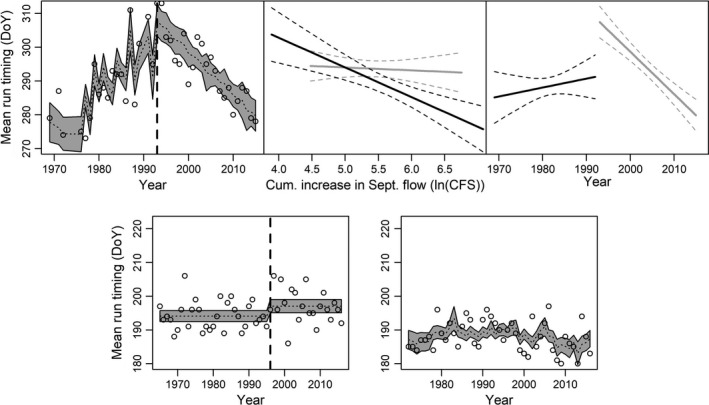
Fits and partial dependency plots for AIC‐selected trend models. Panels a), d), and e) show model‐predicted trends in median dates for Cedar River entry, Baker River arrival, and Ballard Locks arrival, respectively. Shaded areas show 95% confidence intervals, and open circles show observed values. Panel b) shows the partial dependency of Cedar River median entry timing on river flow before 1993 (in black) and after (in gray). Panel c) shows residual temporal trends for the same periods after accounting for the influence of flow. If included in the model, vertical dashed lines indicate break years. In partial dependency plots, dashed lines show 95% confidence intervals. *Y*‐axes for all plots show equal ranges to facilitate comparison of trend magnitude between panels

The most parsimonious models indicated that the temporal trends in timing varied between the Ballard Locks, Cedar River, and Baker River. The timing of sockeye salmon arrival at the Ballard Locks and Baker River changed little between ~1970 and 2016 (Figure 3d,e). For each of these time series, the selection procedure did not identify a single, strongly favored model. Rather, nearly every model had >1% support, and at least three had a > 10% probability of being the best model (Table 3). May water temperature at Race Rocks (reflecting marine conditions on the migration route) was included in all of the highest weighted models for arrival timing at the Ballard Locks. Environmental variables were not included in the highly weighted Baker River models. However, pairwise comparisons of the time series found that median timing of sockeye salmon arrival at the Ballard Locks was moderately correlated with the initiation (i.e., 10% completion) of the Baker Lake run (*r = *0.58; Table 4).

**Table 3:** Summary of model comparisons for temporal trends in arrival of sockeye salmon to the Baker Lake system, reflecting entry into freshwater there; the Ballard Locks, reflecting entry into the Lake Washington system; and the Cedar River, where most Lake Washington salmon are produced

ID#	Baker Lake			Ballard Locks			Cedar River		
	AIC	AIC_w_	Break	AIC	AIC_w_	Break	AIC	AIC_w_	Break
1a	**311.51**	**0.11**	N/A	266.13	0.01	N/A	319.86	0.00	N/A
1b	313.20	0.05	N/A	263.26	0.04	N/A	301.52	0.00	N/A
1c	312.42	0.07	N/A	266.59	0.01	N/A	315.11	0.00	N/A
1d	313.93	0.03	N/A	264.20	0.03	N/A	302.67	0.00	N/A
2a	***310.22***	***0.20***	***1996***	262.90	0.05	2007	310.07	0.00	1979
2b	313.52	0.04	1996	**260.05**	**0.20**	**1995**	292.03	0.00	2004
2c	314.18	0.03	1996	264.03	0.03	2007	295.11	0.00	2010
2d	313.19	0.05	1989	263.92	0.03	1995	383.37	0.00	2008
3a	**311.31**	**0.12**	N/**A**	267.11	0.01	N/A	321.02	0.00	N/A
3b	313.14	0.05	N/A	***259.63***	***0.25***	**N/A**	303.51	0.00	N/A
3c	313.29	0.04	N/A	264.86	0.02	N/A	317.04	0.00	N/A
3d	314.55	0.02	N/A	261.39	0.11	N/A	304.44	0.00	N/A
4a	**311.27**	**0.12**	**1997**	263.13	0.04	1994	284.42	0.00	1993
4b	313.95	0.03	1997	**261.10**	**0.12**	**2013**	***272.00***	***0.82***	***1993***
4c	314.73	0.02	1997	267.76	0.01	1994	287.30	0.00	1993
4d	314.54	0.02	1997	263.16	0.04	2014	**275.00**	**0.18**	**1993**

Note

Models receiving greater than 10% AIC weight are in bold. Lowest AIC models are italics.

**Table 4:** Pairwise comparisons between sockeye salmon run timing metrics at three locations: Ballard Locks (the entry into the Lake Washington basin), the Cedar River (the primary Lake Washington basin spawning area), and Baker Lake (entry into that basin by a geographically proximate and genetically similar population)

	Year	Baker Lake				Cedar River				Ballard Locks		
		10%	50%	90%	Duration	10%	50%	90%	Duration	10%	50%	90%
Baker 10%	0.22											
Baker 50%	0.20	**0.76**										
Baker 90%	0.45	0.67	**0.69**									
Baker dur.	0.39	−0.05	0.21	**0.71**								
Cedar 10%	0.48	0.14	0.05	0.25	0.21							
Cedar 50%	0.07	0.16	0.07	0.08	−0.04	**0.78**						
Cedar 90%	−0.31	0.12	0.07	−0.08	−0.23	0.33	**0.85**					
Cedar dur.	−0.66	0.00	0.02	−0.28	−0.38	−0.48	0.17	**0.67**				
Locks 10%	−0.18	**0.51**	0.25	0.18	−0.26	0.04	0.28	0.38	0.31			
Locks 50%	−0.15	**0.58**	0.26	0.23	−0.26	0.08	0.30	0.39	0.30	**0.88**		
Locks 90%	−0.01	**0.53**	0.28	0.33	−0.07	0.33	0.48	0.45	0.15	0.71	**0.87**	
Locks duration	0.20	0.16	0.10	0.26	0.20	0.41	0.35	0.19	−0.15	−0.17	0.19	**0.57**

Note

Reported values are Pearson correlation coefficients (*r*). 10%, 50%, and 90%—run completion percentiles. Duration—calculated as the difference between 90% and 10% completion dates.

In contrast to the ambiguous model selection results for the Ballard Locks and Baker River arrival timing, trends in Cedar River entry timing were best explained by a single class of models: those including two separate linear trends and the cumulative increase in Cedar River flow during September (Figure 3a). Model 4b (Table 3) received over 80% of the AIC weight, while model 4d which also included average autumn Lake Washington surface temperature received the remaining weight. In both of these models and the other two including multiple linear trends, the break year was consistently identified as 1993 (Table 3). Examination of coefficients from model 4b showed that from 1969 through 1993, higher September flow increases were associated with earlier entry timing, but since 1994, this influence has been greatly reduced (Figure 3b). Conversely, after accounting for environmental change (i.e., a decreasing pattern in September flow; Figure 5), no significant temporal trend was apparent prior to 1994, but since then, median timing has become earlier at a rate of 1.26 days per year (Figure 3c; Table 5). Timing was not highly correlated between the Ballard Locks and Cedar River (*r = *0.30; Table 4).

**Table 5:** Summary of model coefficients for the best‐fit model of changes in Cedar River sockeye salmon spawning timing

Variable	1969–1993		1994–2015	
	Coefficient	*p*‐value	Coefficient	*p*‐value
ln(September flow increase)	−8.80	<0.001	−0.86	0.01
Year	0.25	0.335	−1.26	<0.001
*F = *22.75 on 5 and 36 degrees of freedom. *R* ^2^ = 0.72, *p < *0.001				

#### 3.1.1 Heritability of spawning timing in cedar river sockeye

Regression analysis of standardized offspring and parent spawning timing resulted in separate estimates of realized heritability of this trait in Cedar River sockeye salmon returning at age‐4 and age‐5. For age‐4 returns *h* ^2^ was estimated to be 0.86 (95% CI: 0.62–1.1) and 0.79 (95% CI: 0.5–1.08) for age‐5 returns (Figure 5). Compared with published estimates of *h* ^2^ for salmonid spawning timing, these estimates are high but not unprecedented (Abadía‐Cardoso et al., 2013; Crozier et al., 2011; Dickerson, Willson, Bentzen, & Quinn, 2005). Consequently, we considered a broad range of *h* ^2 ^in our sensitivity analysis.

#### 3.1.2 Artificial selection on spawning timing

Examination of the yearly dates when the weir was in place in the river to collect sockeye salmon for spawning in the hatchery revealed considerable variation, but in general, the very earliest returning salmon tended to arrive before the weir was in place, after which the weir was operating to trap salmon (but by no means all), and toward the end of the season high river flows precluded further trapping so the late arriving fish were not trapped. From the dates when salmon were spawned in the hatchery, we calculated the median spawning date in each year (i.e., the date by which 50% of the annual total number of eggs had been fertilized in the hatchery). These dates advanced by ~1.26 days per year over the hatchery program's operation (1991–2015).

**Figure 4:**
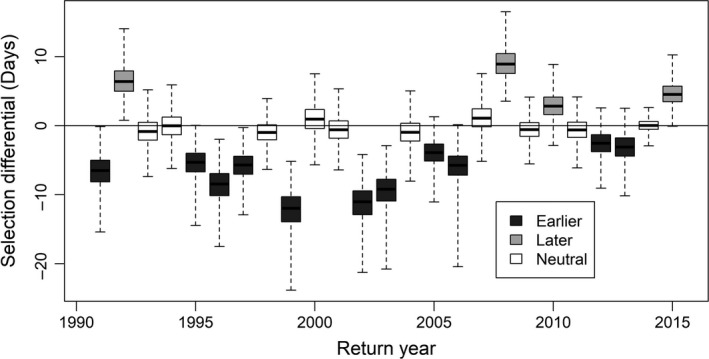
Box plots of annual selection differentials on Cedar River sockeye salmon spawning timing. Heavy lines show medians, boxed areas show interquartile ranges (IQR), and whiskers show full range of Monte Carlo output. Boxes are shaded based on the position of the IQR relative to zero, indicating years in which selection favored salmon that returned earlier (dark boxes) or later (gray boxes) than average, or when selection was neutral (white boxes)

The selectivity of hatchery operations on spawning timing varied in strength and direction between years, but in most years favored earlier spawning fish (Figure 4). Using the point estimates in Table 1 for each parameter, hatchery spawning occurred on average 3.9 days earlier than natural spawning each year. Accounting for the egg‐to‐fry survival advantage of hatchery‐spawned adults, the average selection differential was −3.2 days, and the average expected trait change was −0.95, −1.6, and −2.6 days per generation for *h* ^2^ values of 0.3, 0.5, and 0.83, respectively. The change in spawning timing between 1991 and 2015 resulting from artificial selection can be approximated by multiplying the average expected trait change (excluding those spawned in 2012–2015 for which adults have not yet returned, and any selection will not be realized in offspring timing) by the average number of generations that have experienced artificial selection during this period (5.25, assuming a 4‐year generation time, which is the dominant life history in this population). Given low, medium, and high estimates of heritability, the cumulative effect of artificial selection on spawning timing was estimated to be −5.6, −9.8, and −16.2 days, respectively.

**Figure 5:**
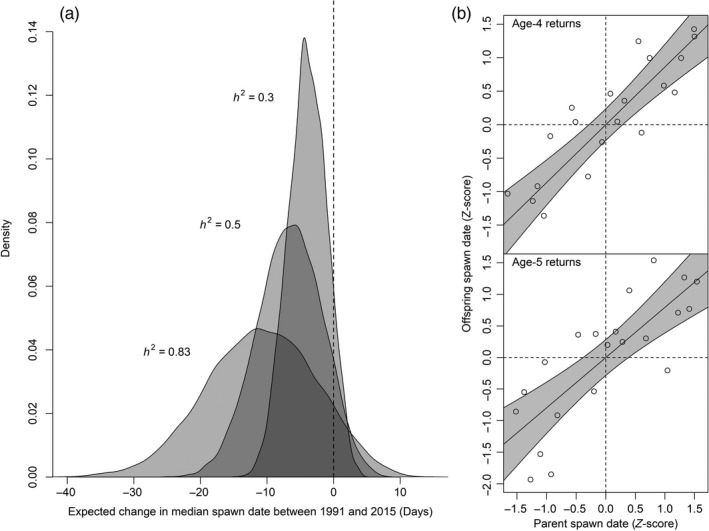
a) Distributions of expected change in median spawning date based on 10,000 Monte Carlo simulations at three levels of heritability (*h* ^2^). Vertical dashed line shows zero expected change; negative values indicate an expected change toward earlier spawning. b) Scatter plots showing the relationships between parent and offspring spawning timing for age‐4 and age‐5 returning Cedar River sockeye which support the highest *h* ^2 ^used in sensitivity analyses. Lines and shaded areas show linear model best fits (*R* ^2^ = 0.79 and 0.86 for age‐4 and age‐5 returns, respectively)

**Figure 6:**
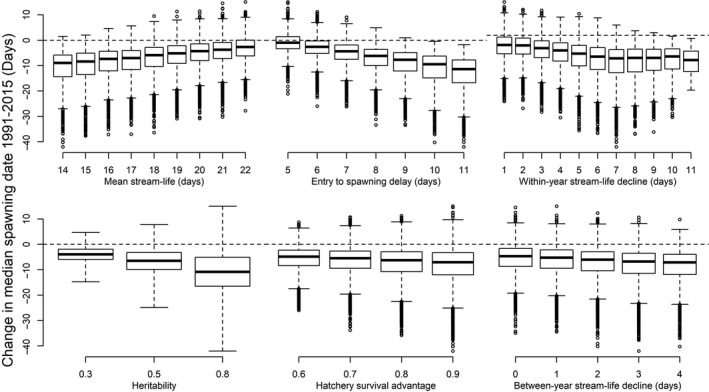
Sensitivity of Cedar River sockeye arrivals model to parameter values. Box plots of the distribution of model outputs given each parameter value. Heavy bars show medians, boxes show the interquartile range, whiskers show the overall range, and open circles show outliers

The Monte Carlo sensitivity analysis allowed us to explore variability in these estimates given uncertainties in model parameters. Figure 5 shows the frequency distributions of artificially induced change in spawning timing between 1991 and 2015, given the ranges of parameter values (Table 1). Regardless of heritability level, more than 90% of parameter combinations resulted in advancing spawning timing. The frequency distributions of the Monte Carlo output were centered on −3.9, −6.5, and −10.8 days for low, medium, and high heritability. Plotting the Monte Carlo output against values of each model parameter allowed us to examine their relative influence. In general, the model output was most sensitive to stream‐life and the delay between arrival and spawning, while higher values for heritability and the hatchery survival advantage tended to amplify the variability of the model output. The effects of each parameter on expected change in spawning timing are shown in Figure 6. The observed change in Cedar River spawning timing between 1991 and 2015 was more than −20 days. A change of this magnitude resulting solely from artificial selection is unlikely unless heritability of spawning date is very high in this population. While 13.7% of parameter combinations produced a shift toward earlier spawning at least this large when *h* ^2^ = 0.83, the proportion fell to 0.3% and 0% in the medium and low heritability scenarios.

## 4 DISCUSSION

The reproductive phenology of Cedar River sockeye salmon has changed markedly over the past five decades. Between 1969 and 1993 river entry and median spawn timing of sockeye salmon became progressively later, a pattern that may be partly explained by a trend toward lower late‐summer flows in the Cedar River. In the early 1990s, the pattern changed substantially, and from 1994 through 2015, the date of median spawning advanced by over three weeks. Our comparative analysis of trends in homeward migration and spawning timing indicated that within‐watershed processes are likely responsible for the majority of observed change because the timing of freshwater entry has remained relatively stable, and little change was observed in the phenology of the Baker Lake (origin) population. Although trends in the Cedar River flow regime explain much of the variation in spawning timing up until the early 1990s, the shift toward earlier spawning that has occurred since is not well explained by environmental conditions. However, the observed shift toward earlier spawning began approximately coincident with initiation of hatchery supplementation. Analysis of hatchery spawn timing relative to the phenology of natural spawners indicated that artificial selection for earlier spawning has occurred in the past, and, depending on the heritability of the trait, can explain a substantial proportion of the observed advance in spawning timing since the mid‐1990s. This was not a case of deliberate selection; rather, the operation of the weir needed to collect the salmon for spawning was not operable late in the fall, owing to high flows. Thus, the latest arriving salmon were unlikely to be spawned in the hatchery, where they would enjoy much higher survival of their embryos than would occur in the river. However, some salmon ascended at the very beginning of the season, prior to the weir's installation, and they too were unspawned. Thus, the hatchery selected, on average, for early spawning but was also to some extent exerting disruptive selection. These findings collectively provide strong evidence that even in an integrated hatchery population, inadvertent selection on timing can induce population‐level changes in phenology.

Although there is strong evidence that artificial selection on reproductive phenology has occurred, it is not clear that this alone can explain the dramatic advance in Cedar River sockeye salmon spawning timing since the 1990s. Our evolutionary model and sensitivity analysis demonstrated that if heritability is close to our estimate of 0.8, then certain combinations of plausible stream‐life parameters can produce advances in spawning timing of greater than three weeks, given observed patterns of hatchery selection. We used this heritability estimate with some caution as it is high compared with previous studies, though not improbably so. At the moderate heritability level (*h* ^2^ = 0.5), both our best estimates of model parameters and the central tendency of Monte Carlo output suggested that the effect of artificial selection has been more modest—on the order of 7 to 10 days—than the observed change of around three weeks. The additional change in phenology might have resulted of altered temperature or flow regimes. However, our analysis of environmental covariates failed to support this hypothesis. Prior to the influence of hatchery supplementation, some aspects of Cedar River flow and Lake Washington temperature were well correlated with Cedar River entry timing, and the trend toward later spawning between 1969 and 1993 was generally consistent with a pattern of warming autumn water temperatures and decline in September freshets. Since 1994, however, relationships with environmental covariates have broken down, likely as a result of the effect of artificial selection on phenology. Furthermore, both theory (Quinn, 2018) and studies of sockeye salmon (e.g., Kovach, Ellison, Pyare, & Tallmon, 2015) and other salmonids (e.g., Warren et al., 2012) indicate that spawning should occur later in response to warming waters; the opposite of the pattern observed since 1994 in the Cedar River. Thus, while we cannot rule the effect of other environmental factors for which we had no data, our findings suggest that inadvertent selection in the hatchery is likely occurring.

The spawning timing phenotype that is ultimately observable (i.e., median spawning date in the population) emerges from a series of complex and interrelated evolutionary, behavioral, and physiological processes. In our study population, spawning timing appears largely independent of ocean processes because, although marine environmental factors do influence the timing of arrival in freshwater, a long delay before spawning combined with a lack of relationship between lake and river entry timing appears to decouple migratory and reproductive processes (Newell et al., 2007). Thus, Cedar River sockeye salmon spawning timing appears to be determined by an underlying, genetic predisposition combined with plastic response to flow, and possibly temperature conditions in the lake and river. Spawning timing can also vary with fish size, age, and spawning location, so long‐term changes in these traits may influence spawning timing (Carlson, Rich, & Quinn, 2004; McPhee & Quinn, 1998). Finally, because sockeye salmon cease feeding prior to freshwater entry, a fixed energy budget is available once the fish are in Lake Washington. It has been suggested that early upstream migration in sockeye salmon may occur if energy reserves are atypically low (Lapointe et al., 2003) or high (Katinić, Patterson, & Ydenberg, 2017). Energy availability can in turn be influenced by many factors including temperatures experienced during migration and staging, or by pathogens. Despite uncertainty regarding the influence of these processes in the Cedar River population, given the selection differentials we observed and high estimated heritability of spawning date in the population, we conclude that unintended artificial selection in the hatchery has contributed substantially to the trend toward earlier spawning since the mid‐1990s.

Although we are unable to directly test the fitness effects of altered reproductive phenology in Cedar River sockeye salmon, several lines of evidence suggest that artificial selection for early spawning may be maladaptive in this population. First, juvenile sockeye salmon that enter Lake Washington from the Cedar River later in the spring on average experience a survival advantage over the earlier migrants of their cohort (Hovel et al., 2019). This is presumably because later migrants are more likely to experience a “match” with favorable growth conditions during the vulnerable period shortly after lake entry (Cushing, 1990). Because sockeye salmon spawn as stream temperatures are decreasing, the effect of earlier spawning is amplified in emerging juveniles. Embryonic development is strongly influenced by temperature, and earlier spawning exposes embryos to warmer water (Murray & McPhail, 1988). Given the average temperature regime of the Cedar River, a one‐week shift in median spawning from mid‐ to early October would advance median emergence timing by over two weeks. The impact of artificial selection on spawning timing is therefore amplified in juvenile phenology. Combined with the relative survival advantage for later lake entry by juveniles, disruption of offspring trophic dynamics is a plausible fitness cost of artificial selection for earlier spawning in this population. Spring lake processes—and therefore optimal juvenile entry timing—are also advancing in response to climate change (Winder & Schindler, 2004), but the change in spawning timing since the 1990s has far exceeded the environmental change.

Artificial selection for earlier spawning may also reduce fitness through increased exposure of adult sockeye salmon to temperatures warm enough to elevate prespawning mortality (PSM) rates. Cedar River sockeye salmon enter Lake Washington prior to peak summer temperatures, hold in the lake's hypolimnion through the summer, and then enter the river and spawn in the cooler fall months (Newell and Quinn 2005). Thermal conditions in the Ship Canal and in Lake Washington have been warming and, in recent years, unexplained mortality has been documented in sockeye salmon spawning in the river (22%–34% annually) and in the hatchery (31%–41% annually: Barnett, Peterson, Yates, & Drobny, 2018). PSM such as this occurs in many salmon populations (Bowerman, Keefer, & Caudill, 2016) but has been rare in the Cedar River (Ames, 2006). The causes of PSM vary, but elevated temperatures can be a contributing factor (Bowerman et al., 2018). Earlier migration into the Cedar River, combined with a general pattern of warming in Lake Washington, may be driving an increase in the rate of PSM. Within‐season patterns of PSM support this idea; the earliest fish have experienced the highest rates of failed spawning. Carcass surveys showed that PSM rates decreased from an average 49% in September, 26% in October, 24% in November, to 13% in December for years 2014–2016 (Barnett et al., 2018). If earlier spawning is associated with lower fitness from either reduced survival of progeny or inability of adults to breed successfully, then artificial selection on this trait will cause a larger proportion of the population to spawn during less favorable times. While the abundance of Cedar River sockeye salmon has declined markedly during the past decade, it is impossible given the available data to attribute any specific portion of declining abundance to artificial selection on phenology. Nevertheless, our results suggest that altered phenology is a plausible contributing factor of population declines.

Artificial propagation may be warranted if there are demographic risks to a population's viability, and in the case of Pacific salmon, has sustained fisheries in cases where habitat loss reduced capacity for natural production (Flagg, 2015; Naish et al., 2007). However, our findings add to a growing body of evidence documenting the challenges in sustainable supplementation of fish populations (Amoroso, Tillotson, & Hilborn, 2017; Araki 2007, 2008; Baskett & Waples, 2013). Hatcheries can reduce genetic diversity (Berejikian & Van Doornik, 2018; Waters et al., 2015), impose artificial selection (McLean et al., 2005; Quinn et al., 2002), and may disrupt processes of natural selection and adaptive evolution (Waples, Beechie, & Pess, 2009). Efforts are underway to improve the genetic management of hatcheries in some regions (Flagg, 2015; McGarvey & Johnston, 2011; Mobrand et al., 2005) but impacts on supplemented populations may occur if wild and hatchery fish cannot be fully segregated (e.g., Seamons, Hauser, Naish, & Quinn, 2012). Our findings further highlight that phenology can be particularly sensitive to inadvertent selection in hatcheries (see also Quinn et al., 2002; McLean et al., 2005; Ford et al., 2006). As with selection on timing in fisheries (Tillotson & Quinn, 2018), selection on timing in hatcheries can result from a number of processes including the natural tendency to spawn the first arriving fish, leaving those at the end to be released into the river if the capacity of the hatchery is filled. In addition, and more germane in the present case, river conditions favor the trapping of early arriving salmon more than those coming later, and thus, the progeny of the early fish is given the benefit of the higher survival rate during incubation in the hatchery. Because the timing of migration and reproduction is a key component of salmon diversity, the management of fisheries and hatcheries should strive to maintain the natural variation in these traits (Tillotson & Quinn, 2018). Furthermore, consideration should be given to how warming waters and changing flow regimes (e.g., Arismendi, Safeeq, Johnson, Dunham, & Haggerty, 2013) might interact with hatchery operations to ultimately shape patterns of selection on supplemented populations.

## DATA ARCHIVING STATEMENT

Data available from the Dryad Digital Repository: https://doi.org/10.5061/dryad.3fc8m7c.

## CONFLICT OF INTEREST

None declared.
